# Book and Pamphlet Notices

**Published:** 1858-06

**Authors:** 


### BOOK AND PAMPHLET NOTICES.

Materia Medica and Therapeutics, with ample illustrations of practice, in all the departments of Medical Science, and very copious notes on Toxicology, suited to the wants of Medical Students, Practitioners and Teachers. A new edition, revised and enlarged. By Thos. D. Mitchell, A.M., M.D., Professor of Materia Medica and General Therapeutics in Jefferson Medical College, and formerly Professor of Chemistry, Materia Medica, and Theory and Practice in the Medical College of Ohio, Transylvania University, and the Kentucky School of Medicine; author of Elements of Philosophy, etc. etc. Philadelphia: J. B. Lippencott & Co., 22 and 24 North Fourth street. 1857. Pp. 820. For sale by Keen & Lee, Chicago, Ill.

Dr. Mitchell says he "has added his own experience on various points, and the best testimony of the great world of physic, with the design of putting a book of real value into the hands of the student and practitioner. Theoretical notions, which are but too often of mushroom stability, have been almost entirely eschewed, and the book is therefore chiefly a volume of facts, quite as reliable as any to be furnished by the profession anywhere."

He says, further, "it is believed that every valuable new application of an old remedy, as well as the desirable uses of agents claimed to be new down to the date of this prefatory note, are here presented so as to set forth their real or apparent worth." Now, while we believe that Dr. Mitchell has done what he says he has in the first part of the above quotation from the preface, we cannot believe his statement in the last; his book is too small to do any such thing as to give all the valuable information the medical world possesses in materia medica. As an instance of what we consider almost an unpardonable deficiency, in one valuable article, he disposes of veratrum viride in eleven lines. He says nothing about its peculiar sedative influence, or its applicability to any disease or class of diseases. How the very prominent and indubitable characteristics of this invaluable remedy could have escaped the attention of Prof. Mitchell we cannot divine.

This work is arranged in alphabetical order, instead of the ordinary classifications of authors on these subjects. The reliable information on the subject of poisons contained in Prof. Mitchell's work is worth four times the price of it, and if for nothing else, every practising physician should possess it. He tells us of those antidotes always at hand (in the West at least) which may be depended upon for poisoning by arsenic, viz; magnesia, charcoal and tobacco. And we think it an item to have several articles upon which we know we may depend, as we are more certain to have one of them on hand. We are rejoiced also to know that the filthy article which *inhabits* the pockets of so many Esculapian votaries may be turned to other accounts than staining carpets and masculine perfumery. He says "finely pulverized charcoal is, as I know from actual experience, capable of accomplishing all that is desirable in this relation." It should be given in large quantities in water or otherwise. Magnesia may be relied upon also with equal quantities, if given freely and largely. The best calcined is preferable, but the carbonate answers quite well. He speaks of tobacco in very high terms as a remedy (not as antidote), and thinks that it produces a kind of compound poisoning, in which the original poisoning quality of both articles are lost. However the explanation of its effects, it certainly cures patients poisoned by arsenic. He relates a case, from Silliman's Journal, of "a young lady, the poison having been taken by mistake, and in a region considerably remote from a physician or an apothecary shop. It was supposed that an emetic would give relief, and as nothing offered excepting tobacco, it was resorted to in the hope of exciting emesis and thus dislodging the poison. A decoction or infusion was prepared, and rather cautiously given, fearing it might act with too much violence. Dose after dose was given, time passed away and no emesis could be had, and the result was that the lady was not seriously hurt. The success of the new remedy led to its employment in other cases with equal success, but no vomiting occurred." It is added, that "more than probably an inveterate chewer could not be saved by the exhibition of any quantity of this article." If it were not for encouraging the use of this vile weed, we would express doubts of this conjecture of Prof. Mitchell. He speaks of the hydrated peroxide of iron also, as do most writers on the subject, but is no better than the two first-named articles.

Contributions to Operative Surgery and Surgical Pathology. By J. M. Carnochan, Professor of Surgery in the New York Medical College, Surgeon-in-Chief to the State Emigrants' Hospital, etc. With Illustrations from Nature. Philadelphia: Lindsay & Blackiston. 1858.

Dr. Carnochan proposes to issue those contributions quarterly, in numbers containing from thirty to sixty pages each, until the series is complete; in all there will be ten. We have received the first, which contains thirty-two pages. It is gotten up in superb style, in quarto size, and illustrated with elegant engravings; but what is better still, it bears the impress of the vigorous originality so characteristic of the author. We quite agree with Dr. Carnochan in the opinion, that "it is from the study of particular facts operative surgery derives its most valuable suggestions, and as there is no limit to the multiplicity of pathological differences, the most experienced practitioner may find himself in the presence of unknown and unforeseen conditions. Hence, it is incumbent on the surgeon to read much as well as to see much, and to extend his knowledge as far as possible beyond the sphere of his own immediate observation."

The number on our table contains a case of amputation of the entire lower jaw; remarks on amputation of the lower jaw; elephantiasis Arabum successfully treated by ligature of the femoral artery, with cases.

No. 2 will be case of exsection of the entire ulna; remarks on neuralgia, with three cases successfully treated by exsection of the second branch of the fifth pair of nerves beyond the ganglion of Meckel.

No. 3.—Case of restoration of the entire upper lip; remarks on the congenital dislocation of the hip joint, with illustrations.

No. 4.—Case of exsection of the entire radius; case of exsection of the three lower fourths of the same bone; remarks on osteo-aneurism, with a case.

No. 5.—Case of amputation of the shoulder joint, for the removal of an osteo-fibro-cancerous tumor of the humerus, with remarks on amputation at this joint; case of penetrating gunshot of the heart.

No. 6.—Case of double congenital dislocation of the hip joint; remarks on double capital operations, with cases; remarks on the comparative merits of the partial amputations of the foot; remarks on amputations through the ankle joint.

No. 7.—Successful removal of a large fibro-cartilagenous tumor growing from the sixth and seventh ribs over the region of the heart; remarks on the treatment of varicose veins of the lower extremities, with cases; remarks on the creation of an artificial joint on the lower jaw, in a case of complete anchylosis at the tempero-maxillary articulation of one side.

No. 8.—Remarks on the operation of double complicated hare-lip; remarks on the etiology of congenital dislocation of the hip joint; remarks on the removal of the first dressings after capital amputations.

No. 9.—Case of encysted sanguineous tumor of the neck successfully removed, with remarks on such formations; remarks on the purulent ophthalmia of large and crowded institutions; case of vesico-vaginal fistula and stricture of the vagina, with formation of two large urinary calculi in the vagina behind the stricture, spontaneons cure of the fistula.

No. 10.—Two cases of amputation at the hip joint; remarks on the anatomy of femoral hernia; case of epilepsy treated by tracheotomy, and wearing of a tracheal tube, with remarks; remarks on the entire restoration of the lower lip, with cases; cases of amaurosis treated with the pomade de Goudret on the sinciput.

These numbers are in the course of preparation, and will form one complete volume, the first of a series. Terms of subscription—each number twenty-five cents, to be paid for on delivery. Published by Lindsay & Blackiston, Philadelphia.

Judging from the number before us, we expect a ready sale.

We have received a slip from Dr. Bennet Dowler, on the methods of preserving bodies for dissection, which is so short and at the same time satisfactory, that we republish the whole. Alcohol is not the best article in which to preserve anatomical specimens as a general rule, and we are glad to extend any information that may enable the profession to dispense with its use in one more respect.

In an article on Medical Schools, in the November number of the *New Orleans Medical and Surgical Journal*, I alluded to a method of preserving bodies for dissection, which I regarded as a discovery of great importance to students of anatomy, and particularly to those of our Southern States, where the climate is so unfavorable to anatomical pursuits. I have had a good many letters from medical gentlemen requesting me to furnish the formula, and I take much pleasure in laying it before your readers, hoping that this important discovery may awaken a new interest in this fundamental branch of our profession.

The following is the formula which I have used in New Orleans with perfect success, and there can be no question that if a subject is properly injected, it will keep as long as desirable. Take two parts, by measure, of muriatic acid, and one part of water, and as much metallic zinc as they will dissolve—use it undiluted. Cut down on the arch of the aorta, and throw in as much of the fluid (according to the size of the subject) as can be injected without excessive force—say from two to four quarts. If it is well done, the muscles all become of a slate color, and the tissues firm. I have now on the table a subject on which I have been demonstrating to my class for fifteen days, and it is entirely free from odor, or other sign of putrefaction.

J. C. Nott, M. D.,
*Professor of Anatomy, University of Louisiana.*

P. S.—The injection should be thrown in gradually, and it is well to wait some minutes between each syringe full of the fluid. A body may be well injected, if done with skill, by the carotid, or femoral artery, but when the apparatus is imperfect it is better to saw the sternum longitudinally, force the chest open, and place the pipe in the arch of the aorta. Bodies are "embalmed" in most of the cities of the United States by a similar process, and I this morning saw more than a gallon of fluid thrown in through the radial artery, at the wrist. The subject was a lady, whose body is to be transported to the North.

[I beg leave to append to the above communication from the distinguished professor of anatomy in the University of Louisiana, the following note concerning the preservation of the human body, during a course of dissections in any climate. If not the newest, it is certainly the cheapest method known. Many years ago, in Virginia, in order to afford my private pupils anatomical facilities with the utmost privacy and security, I was compelled to use the garret of my dwelling, where the heat was great, and the putrefactive process was, consequently, rapid during midsummer. I was able to carry on dissections without hurry and without the putrefaction of the subject in the month of August, as well as in the winter. The means adopted were very simple—namely, when the subject was not under examination, it was kept constantly and completely immersed in a saturated solution of common salt. This method neither alters the natural color of the tissues, nor subsequently interferes with the making of handsome dried preparations when the dissection shall have been completed. Before proceeding to dry an anatomical piece, a little soaking in fresh water may be proper, to prevent any saline incrustations, but this is not necessary generally. No accident to health and but little damage to knives can result from this method of anatomizing. In fact, no climate producing salt and water can be so hot as to render this method inapplicable. The human body can be preserved just as well as pickled pork, beef, fish, and the like. Whether in high or low latitudes, all dissecting rooms which are kept sufficiently warm to be healthful and comfortable to the living, must be unfavorable to the prolonged preservation of the dead, and produce emanations of a deleterious character. BENNET DOWLER.]

